# Effect of Winemaking on the Composition of Red Wine as a Source of Polyphenols for Anti-Infective Biomaterials

**DOI:** 10.3390/ma9050316

**Published:** 2016-04-27

**Authors:** Arianna Di Lorenzo, Nora Bloise, Silvia Meneghini, Antoni Sureda, Gian Carlo Tenore, Livia Visai, Carla Renata Arciola, Maria Daglia

**Affiliations:** 1 Department of Drug Sciences, Medicinal Chemistry and Pharmaceutical Technology Section, University of Pavia, V.le Taramelli 12, Pavia 27100, Italy; arianna.dilorenzo01@universitadipavia.it (A.D.L.); silvia.meneghini01@universitadipavia.it (S.M.); 2 Molecular Medicine Department, Center for Health Technologies (CHT), UdR INSTM, University of Pavia, Viale Taramelli 3/b, Pavia 27100, Italy; nora.bloise@unipv.it (N.B.); livia.visai@unipv.it (L.V.); 3 Research Group on Community Nutrition and Oxidative Stress and CIBEROBN (Physiopathology of Obesity and Nutrition), University of Balearic Islands, Palma de Mallorca, Balearic Islands E-07122, Spain; tosugo@hotmail.com; 4 Department of Pharmaceutical and Toxicology Chemistry, University of Napoli Federico II, Via D. Montesano 49, Napoli 80131, Italy; gctenore@unina.it; 5 Department of Occupational Medicine, Toxicology and Environmental Risks, S. Maugeri Foundation, IRCCS, Via S. Boezio 28, Pavia 27100, Italy; 6 Research Unit on Implant Infections, Rizzoli Orthopaedic Institute, via di Barbiano 1/10, Bologna 40136, Italy; 7 Department of Experimental, Diagnostic and Specialty Medicine (DIMES), University of Bologna, via San Giacomo 14, Bologna 40126, Italy

**Keywords:** red wine, winemaking process, metabolite profiling, polyphenols, anti-infective biomaterials

## Abstract

Biomaterials releasing bactericides have currently become tools for thwarting medical device-associated infections. The ideal anti-infective biomaterial must counteract infection while safeguarding eukaryotic cell integrity. Red wine is a widely consumed beverage to which many biological properties are ascribed, including protective effects against oral infections and related bone (osteoarthritis, osteomyelitis, periprosthetic joint infections) and cardiovascular diseases. In this study, fifteen red wine samples derived from grapes native to the Oltrepò Pavese region (Italy), obtained from the winemaking processes of “Bonarda dell’Oltrepò Pavese” red wine, were analyzed alongside three samples obtained from marc pressing. Total polyphenol and monomeric anthocyanin contents were determined and metabolite profiling was conducted by means of a chromatographic analysis. Antibacterial activity of wine samples was evaluated against *Streptococcus mutans*, responsible for dental caries, *Streptococcus salivarius*, and *Streptococcus pyogenes*, two oral bacterial pathogens. Results highlighted the winemaking stages in which samples exhibit the highest content of polyphenols and the greatest antibacterial activity. Considering the global need for new weapons against bacterial infections and alternatives to conventional antibiotics, as well as the favorable bioactivities of polyphenols, results point to red wine as a source of antibacterial substances for developing new anti-infective biomaterials and coatings for biomedical devices.

## 1. Introduction

Growing evidence suggests that natural antimicrobial substances can be used in different fields such as food products (*i.e.*, food preservatives and components for active, intelligent, and environmentally friendly packaging), cosmetic products (*i.e*., cosmetics for oral hygiene, as toothpastes and mouthwashes), medical devices, and antimicrobial drugs. With regard to the latter issue, infections are the leading causes of morbidity and mortality worldwide. According to World Health Organization reports, there were more than 55 million deaths worldwide in 2011 with infection responsible for one-third of all deaths [1]. The long-term exposure to antibiotics has led to the antibiotic resistance of microorganisms; therefore, in recent years, much attention has been paid to the discovery and development of new natural antimicrobial agents that might act against resistant microorganisms [2,3].

Considering that biomaterial research is one of the most important and fastest-growing fields of modern food sciences and medicine, natural products—especially from plant origin—could be novel and interesting candidates for biomaterial applications. Several antimicrobial edible films have been produced to minimize growth of pathogenic microorganisms, including those based on proteins (wheat gluten, casein, and whey protein [4,5], polysaccharides (chitosan) [5], and lipids (acylglycerols)) [6]. In recent years, there has been increased interest in adding antimicrobial agents to medical devices. The aims of the addition are the reduction or prevention of a device-related infection, and the reduction or inhibition of their bacterial colonization. For instance, antimicrobial substances (*i.e.*, thyme oil) have been incorporated into chitosan films for potential applications in wound healing [7].

Plant foods have attracted great interest as antimicrobial substance sources. Among the secondary metabolites occurring in plants, polyphenols, terpenoids, alkaloids, lectins, polypeptides, and polyacetylenes are known to be antimicrobial agents. Most of these metabolites are safe and show negligible toxicity and side effects. Green tea, cranberry, and cocoa are considered the most promising plant sources [8,9,10,11]. Many investigations showed that grapes and related products (grape juice and wine) resulted in exerting antibacterial activity against a wide range of bacteria because of the high content of polyphenols [12,13].

Wine, mainly fermented from *Vitis Vinifera* L. grapes, is a widely consumed beverage due to its pleasant sensory properties. Grape berries and their wines are characterized by high levels of polyphenols, a large family of plant secondary metabolites considered responsible for wine quality and its positive effects on human health [14]. The phenolic quali- and quantitative composition of red wine is influenced by different factors, such as grape variety, vineyard location, climatic condition and sun exposure, winemaking process and wine storage [15]. Red wines, made from dark-skinned grape varieties, generally contain up to 3500 mg/L of phenolic substances including non-flavonoids and flavonoids, the latter group representing an important percentage of phenolic compounds, with amounts ranging from 1000 to 1800 mg/L [16]. The main common representative classes of flavonoids are flavonols, dihydroflavonols, anthocyanins, proanthocyanidins, and flavan-3-ols. Anthocyanins and proanthocyanidins mainly accumulate in berry skin, and are responsible for sensory properties (*i.e*., color, astringency, bitterness, and aroma) and for chemical stability against oxidation. Moreover, tannins are able to form complexes with saliva proteins, conferring astringency and structure to the final beverage. Non-flavonoid compounds include hydroxycinnamic and hydroxybenzoic acids, and phenolic acids which reach concentrations up to 200 mg/L in red wines. Other minor non-flavonoid components are volatile phenols, responsible for off-flavors, and stilbenes, with *trans*-resveratrol recognized as one of wine’s characteristic components [17].

Red wine is traditionally derived from crushing and destemming grapes, with the obtained must remaining in contact with skins and seeds for a greater or lesser period: in this way, the dissolution of the coloring substances of the marc, and possibly of the stalks, is aided by the presence of alcohol produced during fermentation. During the fermentative process, wine is moved from one barrel to another (racking), until the end of both alcoholic and malolactic fermentation.

Nowadays, traditional winemaking has been improved thanks to technological innovations allowing for the production of better quality wines as well as process automation [18]. Moreover, depending on the winemaking method, it is possible to obtain wines with different specific sensory properties and different amounts of bioactive compounds. The addition of co-pigments before prolonged fermentative maceration, generally hydroxycinnamic acids, hydroxycinnamoyltartaric acids and flavonols, can, for example, be used to improve wine color stability and to change its tone from red-purple to intense red. During the maceration, wine anthocyanins come into contact with co-pigments, converting themselves from monomers to polymers [19]. Another winemaking procedure used to improve phenolic content and sensory properties is the addition of condensed tannins derived from grapes. Tannins prompt an increase in anthocyanin stabilization through the formation of polymeric pigments, providing an improvement in color intensity and wine antioxidant and radical scavenging properties [20]. Moreover, a novel winemaking technique consists in leaving yeast lees in the wine after alcoholic fermentation. This procedure induces sensitive changes in wine aroma, because lees have the capacity to adsorb and release certain volatile compounds [21]. Several studies report the effects of wine micro-oxygenation, the continuous addition of small quantities of oxygen to wine. It has become a useful tool for increasing wine color stability, improving its sensorial properties by reducing astringency and increasing fruity and spicy flavors, and for accelerating tannin polymerization [22,23]. Finally, cryomaceration is widely used in the production of white wines and has recently been introduced among red winemaking treatments. This procedure allows for a higher extraction of phenolic compounds contained in grape skins and of primary aroma. The yield of pre-fermentative cold maceration is influenced by parameters that describe the process itself, such as the working temperature (usually ranging from −5 to 5 °C), the use of cryogenic gas (N_2_ or CO_2_) instead of refrigerator groups and the duration of the cryomaceration period. Depending on these parameters, cryomacerated red wines usually show a higher aroma intensity and higher stability in their taste properties, a better quality profile and a higher content of flavonols (*i.e*., quercetin), but a lower content of anthocyanins, catechins and resveratrol [24,25].

A growing body of evidence suggests that moderate intake of red wine exerts protective effects on human health, strongly related to its polyphenolic composition [26]. Polyphenols are well-known antioxidants and free-radical scavengers. Moreover, they can inhibit some transcriptional factors and can modulate enzyme activity and metabolic pathways, thus exerting health benefits with particular regards to chronic degenerative diseases such as cardiovascular pathologies, cancer and diabetes and its complications (*i.e*., diabetic retinopathy) [26,27,28,29,30,31,32,33].

Recent studies show that a moderate consumption of red wine is also related to the protection of oral health. In particular, Daglia *et al.* tested the antibacterial activity of two commercial wines against oral streptococci responsible directly (*S. mutans*) or indirectly (*S. salivarius*) for the development of caries, and against *S. pyogenes* responsible for pharyngitis [12,34,35]. The tested red and white wines were active against tested strains. This activity was mainly ascribed to the presence of organic acids (*i.e.*, lactic, malic, succinic, tartaric, citric and acetic). Moreover, the same research group showed the *in vitro* and *ex vivo* anticaries activity of red wine, by focusing on its antibiofilm and anti-adhesive properties [36]. Dealcoholized red wine, and especially its proanthocyanidins, was shown to strongly interfere with the adhesion of *Streptococcus mutans* to saliva-coated hydroxyapatite (sHA) beads, promoting its detachment from sHA and powerfully inhibiting *in vitro* biofilm formation. In addition, the ability of red wine to inhibit *ex vivo* *S. mutans* biofilm formation on the occlusal surface of natural human teeth was also demonstrated. The same results were achieved by a more recent research that reported that red wine exerted antimicrobial properties in a biofilm model of supragingival plaque, integrated with five bacterial species (*i.e.*, *Actinomyces oris*, *Fusobacterium nucleatum*, *Streptococcus oralis*, *Streptococcus mutans* and *Veillonella dispar*) commonly associated with oral disease [37].

Several investigations on oral disease have been conducted around the world in the last decades because these diseases (such as dental caries and periodontal diseases) are major health problems that influence quality of life and are linked to systemic pathologies, such as cardiovascular diseases, rheumatoid arthritis, osteoporosis, and osteomyelitis [38,39,40,41]. Despite the suggestion from the World Health Organization that dental caries prevalence has been declining around the world and in developed countries in particular, caries is still the most widely spread non-communicable disease in the world. Moreover, dental caries represents an important economic issue due to the higher healthcare costs of therapies [42]. Several synthetic compounds are commercially available for oral health and dental caries treatment. Nevertheless, these substances can alter the oral microbiota and are responsible for side effects (*i.e*., diarrhea and tooth staining). In addition, these products induce uncommon infections and antimicrobial resistance, which represents a worldwide threat to global public health due to new mechanisms of resistance which continue to emerge, strongly increasing the risk of spread of resistant diseases [43]. Alternative prevention and treatment options for dental caries should be put in place to further reduce caries around the world, particularly in developing countries, which need new safe, effective and economical products for the treatment of oral disease. Phytochemicals represent a possible source of effective, cheap and safe anticaries agents and seem to be a suitable alternative [44].

Even though changes in phenolic composition during the winemaking process have been previously investigated, few studies are available to date on the monitoring of the functional properties of wine during the various steps of the winemaking process.

Most of the existing studies are related to the evaluation of antioxidant activity by means of different methods (*i.e*., FRAP, DPPH, oxigen radical absorbance capacity (ORAC) tests) [45,46,47,48]. The aim of this research is to gather combined data on the determination of the chemical composition of wine (metabolic profiling, phenolic and anthocyanin contents) and the evaluation of its antibacterial property against oral pathogens during the course of the winemaking, starting from the crushed grapes through to the end of fermentation. The most promising step of winemaking, during which wine possesses the highest antibacterial activity, will be identified to obtain extracts useful for the preparation of oral health products active against diseases induced by the tested bacteria.

## 2. Materials and Methods

### 2.1. Chemicals and Reagents

Cyanidin-3-glucoside and gallic acid were purchased from Phytolab GmbH & Co. KG (Vestenbergsgreuth, Germany). High Performace Liquid Chromatography (HPLC)-grade water was obtained from a LC-Pak™ Millex system (Millipore Coorporation, Billerica, MA, USA). Methanol was mass spectrometer-grade (Panreac Quimica, Barcelona, Spain). Formic acid 1.0 M and Folin-Ciocalteau’s reagent were purchased from Sigma-Aldrich Chemical Company (St. Louis, MO, USA). Na_2_CO_3_, potassium chloride, sodium acetate, HCl 37% and glacial acetic acid were provided from Carlo Erba (Milan, Italy). Tryptic soy broth (TSB) was purchased from Difco Laboratories Inc. (Detroit, MI, USA). (4,5-dimethylthiazol-2-yl)-2,5-diphenyltetrazolium bromide (MTT) was obtained from Sigma Aldrich (St. Louis, MO, USA).

### 2.2. Wine Samples and Winemaking Process

Wine samples from a 2015 vintage, made from Croatina and Barbera *V. Vinifera* L. grapes grown in the Oltrepò Pavese region, were obtained from the Italian Oltrepò winery “I Doria di Montalto” (Montalto Pavese, Pavia, Italy). Croatina wine samples were collected at the grape-crushing step (30 September 2015), at each fermentative tank transfer, and at the end of fermentation (30 October 2015). All wine samples, their collection date, the winemaking stage and the abbreviation used in this paper are listed in Table 1.

Croatina wine is obtained through a procedure designed and developed by “I Doria di Montalto” winery oenologists, consisting of a hyperoxigenation of must during fermentation and a micro-oxigenation during the post-fermentative steps. In brief, during grape crushing, 0.1 g of K_2_S_2_O_5_ per kg of grapes is added to avoid anthocyanin oxidation in the aqueous medium. In the meantime, 0.1 g/kg of *Saccharomyces Bayanus* and 0.4 g/kg of yeast nutrients are added to start must fermentation. Next, 48 h after the beginning of alcoholic fermentation in glass-lined concrete tanks, the temperature is controlled to not exceed 27 °C. Subsequently, another 24 h later, the addition of yeast-assimilable nitrogen (YAN), at a level of 180 units YAN, stops the alcoholic fermentation. Then, 7 days after the beginning of fermentation, grape marc is removed from the must and undergoes a harder pressing at 1 atm: the obtained wine, named Croatina Torchiato, was analyzed at three different steps of this winemaking process. The Croatina wine is then decanted for 5 days, to allow malolactic fermentation due to *Oenococcus Oeni* and *Leucococcus* bacteria.

A Barbera wine was included among the samples, since it was used to dilute Croatina wine to obtain the red wine named “Bonarda dell’Oltrepò Pavese D.O.C. (controlled designation of origin)”, another market product (70% Croatina/30% Barbera), in conformity with the strict D.O.C. production regulation. Two other wine samples were collected before and after SO_2_ addition (50 mg/L of wine).

Wine samples were collected into 20 mL plastic tubes and stored at −20 °C prior to analyses. Each sample was divided into two aliquots: the first was submitted to spectrophotometric assays (*i.e*., Folin-Ciocalteau’s and pH-differential method assays) and HPLC, coupled to photodiode array and ion trap mass spectrometer detectors (HPLC-PDA-ESI-MSn), analysis; while the second one was dealcoholized under vacuum at room temperature and submitted to microbiological tests, after being restored to the initial volume with Millipore-grade water.

### 2.3. Total Polyphenol Content

Total polyphenol content (TPC) was determined through Folin-Ciocalteau’s method, using gallic acid as standard [49]. In brief, the 0.1 mL wine samples (properly diluted with water in order to obtain an absorbance value within the linear range of the spectrophotometer) underwent an addition of: 0.5 mL of Folin-Ciocalteau’s reagent and 0.2 mL of an aqueous solution of Na_2_CO_3_ (20%; w/v %), bringing the final volume to 10 mL with water. After mixing, the samples were kept in the dark for 90 min. After the reaction period, the absorbance was measured at 750 nm. Each wine sample was analyzed in triplicate and the concentration of total polyphenols was calculated in terms of gallic acid equivalents (GAE), according to the following calibration curve: Absorbance (Abs) = 0.0014 concentration (μg/mL) + 0.0756 (R^2^ = 0.9989), obtained from the analyses of gallic acid solutions ranging from 10 to 700 μg/mL.

### 2.4. Total Monomeric Anthocyanin Content

The total monomeric anthocyanin (TMA) content was determined using the pH-differential method proposed by Lee *et al.* [50]. In brief, after the identification of the proper dilution factor, wine samples were diluted with 0.025 M potassium chloride buffer at pH 1.0 and with 0.4 M sodium acetate buffer at pH 4.5. Sample absorbance was read at 520 nm and 700 nm in each buffer. TMA concentration was calculated as its cyanidin-3-glucoside equivalent (CGE) using the following equation: (CGE, mg/L): (A × MW × DF ×10^3^)/(*ε* × l), with A = (A_520_ − A_700_)_pH1_ − (A_520_ − A_700_)_pH4.5_; where MW is cyanidin-3-glucoside molecular weight (MW = 449.2 g/mol), DF is the dilution factor; l is the path length cell (1 cm); *ε* is the cyanidin-3-glucoside molar absorptivity coefficient (26,900 L/mol cm); A_520_ and A_700_ are sample absorbances read at 520 and 700 nm, respectively, 10^3^ a conversion factor (from g to mg). Data are provided as a mean of three independent measurements ± standard deviation (SD).

### 2.5. HPLC-PDA-ESI-MSn Analysis

The chromatographic analyses were performed using a Thermo Finnigan Surveyor Plus HPLC apparatus equipped with a quaternary pump, a Surveyor UV-Vis photodiode array (PDA) detector, and a LCQ Advantage max ion trap mass spectrometer (all from Thermo Fisher Scientific, Waltham, MA, USA), coupled through an electrospray ionization (ESI) source. Separation was achieved on a Synergi Fusion RP18 80A (150 × 4.6 mm; 4 μm) column, protected by the corresponding guard column, both from Phenomenex, Torrance, CA, USA, operating at 25 °C. A gradient elution was executed with water, using 0.1% formic acid as mobile phase A and methanol as mobile phase B, at a flow rate of 0.3 mL/min. After a 5 min isocratic step at 2% B, elution was started with a linear gradient of B from 2% to 40% in 100 min and from 40% to 100% in 15 min; 100% of B was maintained for 10 min, then B was linearly lowered to 2% in 5 min and the column was re-equilibrated for 15 min. The sample tray temperature was set at 4 °C and the injection volume was 5 μL. The chromatogram was recorded at *λ* = 254, 280, 520 nm and spectral data were collected in the range of 200–800 nm for all peaks.

HPLC-ESI-MS/MS data were acquired under positive and negative ionization modes, using the Xcalibur software. With this aim, the ion trap operated in full scan (100−2000 *m*/*z*), data-dependent scan and MSn modes; in fact, when greater discrimination was required, additional targeted MS^2^ and MSn experiments were performed on selected pseudomolecular ions. To optimize MS operating conditions a preliminary experiment was performed: 10 μg/mL cyanidin-3-glucoside (H_2_O/MeOH:50/50 with 0.1% formic acid) and 10 μg/mL gallic acid (H_2_O/MeOH:50/50 with 0.1% formic acid) solutions were directly infused in the ESI interface at a flow rate of 25 μL/min into the mass spectrometer. The optimized conditions were as follows: sheath gas 60, capillary temperature 220 °C, auxiliary gas 25 and 20, spray voltage 4.5 and 5 kV, capillary voltage −26.13 V and 35 V, respectively, for negative and positive ionization mode.

### 2.6. Bacterial Strains

The following streptococcal strains were used to assess the antibacterial activity of the 15 wine samples: *Streptococcus mutans* 35176 (*S. mutans* 35176), *Streptococcus* *salivarius* 11878 (*S. salivarius* 11878) and *Streptococcus* *pyogenes* BIO1926 (*S. pyogenes* BIO1926). *S. mutans* 35176 and *S. salivarius* 11878 were obtained from the Culture Collection, University of Gothenburg (CCUG, Goteborg, Sweden), while *S. pyogenes* BIO1926 was kindly provided by Roberta Migliavacca (University of Pavia, Pavia, Italy). All bacteria strains were cultured in Tryptic Soy Broth (TSB) at 37 °C in the presence of 5% CO_2_. These cultures were statically incubated at 37 °C under aerobic conditions and reduced to a final density of 1 × 10^10^ cells/mL as determined by comparing the optical density (OD_600_) of the sample with a standard curve relating OD_600_ to cell number.

### 2.7. Evaluation of Minimum Inhibitory Concentration (MIC)

To determine the minimum inhibitory concentrations (MICs) of the wines on the bacteria strains chosen, the standard method of microbroth dilution, as described by the Clinical and Laboratory Standards Institute (CLSI, formerly National Committee for Clinical Laboratory Standards (NCCLS)) [51] was applied with minor modifications. In brief, a liquid TSB medium containing increasing concentrations of wine was inoculated with a predefined number of cells (approx. 10^4^ CFUs/mL) in 96-well microtiter plates. Each plate also included a positive and a negative control. As the addition of wine to media changes the medium color and turbidity, blank samples (medium with the appropriate concentration of wine without bacteria) were also prepared. The value obtained from the blank sample was subtracted from the value obtained for sample turbidity, taking into account the concentration of wine. The turbidity of the medium was measured by a microplate reader (BioRad Laboratories, Hercules, CA, USA) at 600 nm. The minimum inhibitory concentration (MIC) of wine was considered to be the lowest concentration which completely inhibited bacterial growth (when the turbidity of bacterial culture with wine equaled the turbidity of the blank sample). To further confirm the results from the microbroth dilution method, the same density of all bacteria strains seeded in the same conditions and the MIC values were also evaluated by the MTT method as previously described [52].

### 2.8. Statistical Analysis

Statistical analysis was carried out with a statistical package for the social sciences (IBM SPSS 21.0 for Windows). Results were expressed as means ± SE, and *p* < 0.05 was considered statistically significant. The statistical significance of the data was assessed through one-way variance analysis (ANOVA). When significant differences were found, Bonferroni *post hoc* testing was used to determine the difference between the groups involved.

## 3. Results and Discussion

**Figure 1:**
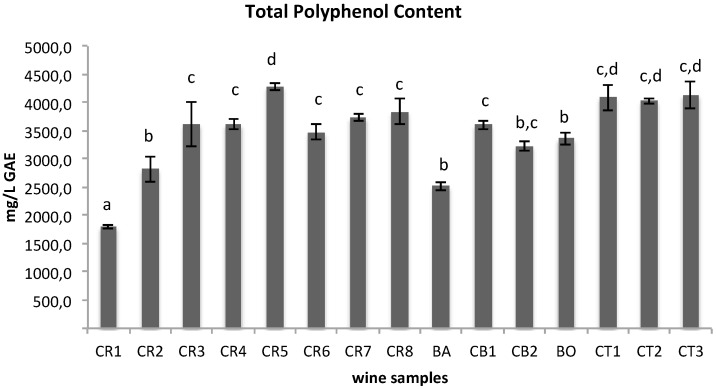
Total polyphenol content of the 15 wine samples by Folin-Ciocalteau’s assay. Data (mg/L GAE) are expressed as the mean ± SD (*n* = 3); different letters indicate statistically significant differences (*p* < 0.05) between samples.

**Figure 2:**
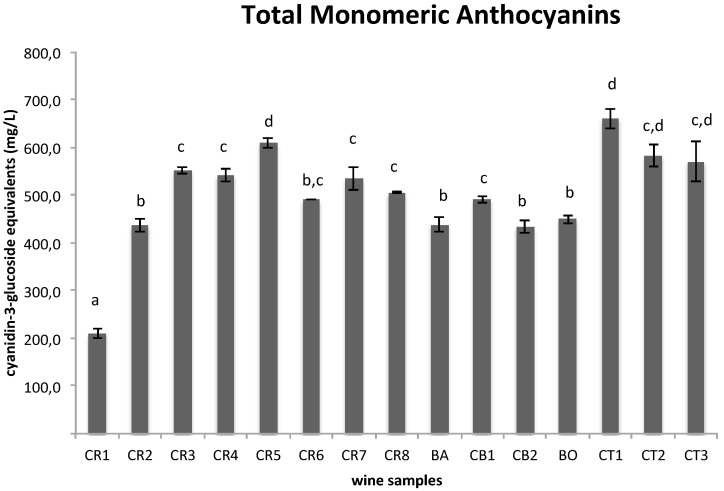
Total monomeric anthocyanin content of the 15 wine samples obtained by pH-differential method assay. Data (mg/L CGE) are expressed as the mean ± SD (*n* = 3); different letters indicate statistically significant differences (*p* < 0.05) between samples.

The first step of this investigation was to evaluate the chemical composition of 15 wine samples obtained as described in Table 1. The samples were submitted to Folin-Ciocalteau’s assay to determine total polyphenol content (TPC), to a pH-differential assay to determine total monomeric anthocyanin content (TMA), and to RP-HPLC-DAD-ESI-MSn analysis to determine their metabolite profiling. According to our data (Figure 1 and Figure 2), TPC and TMA rapidly increase from grape crushing (sample CR1) to the alcoholic fermentation (sample CR3) (from 1.80 to 3.62 g/L GAE and from 210.70 to 550.30 mg/L CGE, respectively, with *p* < 0.05 between the three samples CR1, CR2 and CR3). During the initial stages of winemaking, the increasing extraction of polyphenols and anthocyanins is due to the maceration of grape skin and seeds occurring in the must and to the formation of ethanol during fermentation, which enhances the solubilization of these polyphenolic compounds. From sample CR4 to CR8, which span the duration of the malolactic fermentation period, TPC and TMA values did not change significantly overall (*p* > 0.05). However, Barbera wine had a lower TPC and TMA content (2.5 g/L GAE and 437.5 mg/L CGE, respectively, with a *p* value less than 0.05 between CR8 and BA samples), but after the dilution of Croatina with Barbera (sample CB1) (70% Croatina and 30% Barbera), polyphenol and anthocyanin content did not change significantly (*p* > 0.05, if compared to CR8). A moderate decrease was registered after the addition of SO_2_ for both TPC and TMA (*p* < 0.05, between CB1 and CB2). A recent study compared the effects of two different doses of SO_2_ (50 mg/kg and 100 mg/kg) on red wine phenolic content, demonstrating that only the higher dose of SO_2_ (100 mg/kg) protects polyphenols from oxidation, resulting in higher phenolic content [53]. As far as TMA is concerned, this reduction could be attributed to the reaction between the added SO_2_, present as HSO_3_^−^ in acidic medium, and the flavylium cation causing a decrease of the colored fraction and thus TMA value as reported in the literature [54].

At the end of fermentation (BO), the wine reached a polyphenolic and anthocyanin concentration (3.36 g/L GAE and 443.4 mg/L CG3, respectively) comparable to those determined on average for different *Vitis vinifera* L. cultivar red wines at the same stage [46,47,55]. As far as Croatina Torchiato wine samples are concerned, the TPC and TMA values registered for the first sample, obtained from pressing, were much higher than those of other samples (*p* < 0.05), reaching an average concentration of 4.1 g/L GAE and 659.6 mg/L CGE, respectively. This increase could be explained by the technical procedure to which marc is submitted. In fact, the harder pressing facilitates the extraction of polyphenolic components and anthocyanins from grape skin and seeds. The alcoholic fermentation of the CT1 sample induced a decrease in the TMA values (*p* < 0.05), while TPC turned out to be stable.

**Figure 3:**
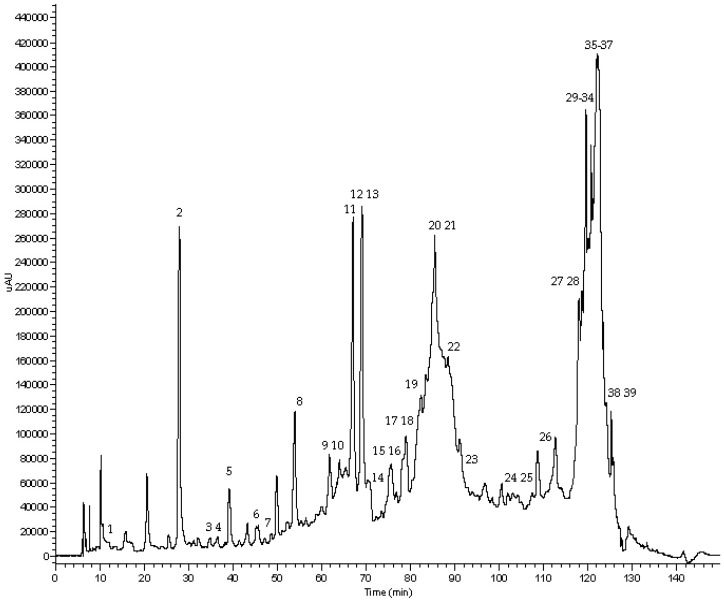
Chromatographic profile of CR5 sample acquired at 280 nm.

RP-HPLC-DAD-ESI-MSn analysis revealed the presence of 39 compounds, listed in Table 2, that were identified according to their chromatographic behavior, their UV-Vis, MS and MS^2^ spectra, when compared with literature data. As an example, we reported the chromatographic profile, acquired at 280 nm, of sample CR5 (Figure 3).

The identified compounds consist of: (a) a stilbenoid (resveratrol-hexoside); (b) six flavonols (kaempferol-hexoside, laricitrin-hexoside, myricetin-hexoside, quercetin-hexoside, quercetin-glucuronide, syringetin-hexoside); (c) four flavan-3-ols (epicatechin, catechin, gallocatechin and a gallocatechin derivative); (d) eight tannins (galloylglucose, three procyanidin dimers, four procyanidin trimers); (e) 18 anthocyanins (peonidin-hexoside, peonidin-3-*O*-(C6-coumaroyl)-hexoside, petunidin-3-acetylhexoside, petunidin-hexoside, petunidin-3-(6-*O*-coumaroyl)-hexoside, delphinidin-hexoside, delphinidin-glucuronide, delphinidin-3-acetylhexoside, cyanidin-hexoside, cyanidin-6-*O*-coumaroylhexoside, malvidin-hexoside, malvinidin-3-acetylhexoside, malvidin-3-coumaroylhexoside, malvidin-3-hexosylacetaldehyde, malvidin-3-hexosylpiruvate, malvidin-3-*O*-coumaroylglucoside pyruvate, malvidin-3-*O*-glucosyl-8-ethyl-epicatechin, carboxypyrano-malvidin-3-coumaroylglucoside); (f) 2 organic and benzoic acids (citric acid, gallic acid).

We will proceed to describe these compounds in greater detail. Resveratrol is a stilbenic compound, characteristic of *Vitis Vinifera* L. grapes and wine, mainly present in its glycosilated forms. It possesses well-known antibacterial, antifungal and antiviral activities [56]. The analysis allowed the identification of resveratrol-hexoside in all the analyzed samples, with the exception of BA in which it was not detectable.

Among flavonols, which exhibit a moderate antioxidant effect and are involved in the co-pigmentation process with anthocyanins during winemaking, kaempferol, quercetin, myricetin and their derivatives are compounds representative of red wine [57]. Indeed, all the analyzed wines showed the presence of kaempferol-hexoside, myricetin-hexoside, quercetin-glucuronide and quercetin-hexoside, with the latter only absent in the BA sample (Table 3). Moreover, the 3’,5’-dimethoxyl derivative and the methoxylation product of myricetin-hexoside, respectively known as syringetin-hexoside and laricitrin-hexoside, were identified in all samples with few exceptions: in fact, syringetin-hexoside was not detectable in the BA sample and laricitrin-hexoside was not detectable in the CR1 sample, suggesting greater formation throughout the winemaking process.

Tannins confer astringency and bitterness to wine by forming complexes with saliva proteins, and have demonstrated antimicrobial activity, including activity against oral pathogens [58]. Three procyanidin dimers and three procyanidin trimers were identified in all 15 samples (Table 3). A fourth procyanidin trimer, found in the CR7 sample, is formed during the winemaking process by condensation reactions of catechin and epicatechin monomers. The presence of galloylglucose was also detected in the third stage analyzed (sample CR3), suggesting higher formation during the fermentative process, though it was not identified in samples BA and CT3.

Flavan-3-ols (*i.e*., epicatechin, catechin and their derivatives) are mainly contained in the solid parts of grape berries (skins and seeds) and possess many healthy properties such as antioxidant and antibacterial effects [59,60]. Among these flavan-3-ols, catechin and gallocatechin derivatives were identified in all samples, while epicatechin and gallocatechin were not detected in samples CR1 and CR2 or in samples CT1, CT2 and CT3 (Table 3), suggesting that the contact between must, skins and seeds allows for an almost complete extraction of these components from the grapes during alcoholic fermentation, as previously reported by Sun *et al.* [45].

Anthocyanins are hydrosoluble pigments representative of red grape varieties and are involved in the formation of co-pigments with flavonoids during winemaking, especially flavan-3-ols and flavonols [57]. Among the 18 anthocyanins identified (Table 4), 14 of them were found in all the samples, ensuring the deep ruby color that characterizes wine made from Croatina *V. Vinifera* L. grapes. Peonidin-hexoside and peonidin-3-*O*-(C6-coumaroyl)-hexoside were identified in trace in all samples. Among delphinidins, delphinidin-3-acetylhexoside was revealed in trace from CR1 to CR6, suggesting that the formation of this acetyl-derivate occurs during fermentation. This data is confirmed by the absence of delphinidin-3-acetylhexoside in samples CT1 and CT2. Petunidin-3-acetylhexoside and petunidin-hexoside, petunidin-3-(6-*O*-coumaroyl)-hexoside were identified in trace in all samples. Among the eight malvidin derivatives, only carboxypyrano-malvidin-3-coumaroylglucoside was not identified in samples CT1, CT2 and CT3, suggesting complete extraction from the first grape crushing.

Finally, gallic acid is an ubiquitarious hydroxybenzoic acid, mainly obtained in wine by the hydrolysis of gallate esters, such as epicatechin gallate. Many healthy properties are ascribed to gallic acid, including antioxidant, neuroprotective and antimicrobial effects [61,62,63]. This non-flavonoid compound was identified in all samples, with the exception of CR1 (Table 3).

The presence of gallic acid in sample CR2 is in agreement with results obtained from the identification of flavan-3-ols, suggesting a relationship between its presence in wine and the hydrolysis of flavan-3-ol gallate derivatives. One organic acid, citric acid, was detected in all samples (Table 3), while other organic acids (*i.e*., lactic, malic and tartaric acid) were not detected, probably because their concentration in the analyzed wines was below the detection limit of the instrument.

As stated above, among the 39 compounds occurring in wine samples, many possess well-documented antibacterial activities [60]. Thus, the subsequent step was to evaluate the antibacterial properties of all wines obtained during the winemaking process against *S. mutans*, *S. salivarius* and *S. pyogenes*. These bacterial strains were chosen for the study with the specific end of assessing the action of wine against different streptococci of the oral cavity, directly or indirectly related to caries development [34,35]. All wine samples were dealcoholized before microbiological tests, with the aim of excluding ethanol interference on streptococcal growth. The estimated MIC values obtained from the dealcoholized beverages are summarized in Table 5. Overall, each wine displayed antibacterial activity against all of the tested pathogenic bacteria. However, some remarkable findings were obtained. Sample CR1 had the highest MIC (60%) of all sampled wines against all tested bacteria strains, with wines collected during subsequent steps of the winemaking process having MICs ranging from 40% to 20%. From sample CR3 to CR8, the MIC values did not change significantly (20% in *S. mutans* and *S. salivarius* and 40% in *S. pyogenes*). Some differences were found across the different species of bacteria, suggesting that the antibacterial effect has a strain-dependent component. Indeed, whereas comparable MIC values were measured in *S. mutans* and *S. salivarius* strains (40% for CR2 wine and 20% from CR3 to CR8), higher MIC values were obtained against *S. pyogenes* (40% from CR2 to CR8). In contrast to this, for the samples falling between BA and BO (from 40% to 20%) all the bacteria strains gave similar results, as was the case for the Croatina Torchiato (CT) wine samples (20%). In summary, these preliminary results show that the initial phase of the fermentation process affects the antibacterial properties of the Croatina, as evidenced by a decrease in the minimal inhibitory concentration from that of CR1. Nevertheless, throughout the progression of fermentation (from alcoholic to malolactic fermentation) the antibacterial activity of the wines does not change significantly, suggesting that the fermentation process does not influence the antimicrobial activity of the wines. Finally, our results also show that this antibacterial effect is still persistent after marc pressing, as demonstrated by the MIC values determined in Croatina Torchiato samples (20% for all bacteria strains).

We found a positive correlation of note between the antibacterial activity and the antioxidant concentration: an enhancement of antioxidants was associated with an increase in antibacterial activity of the same samples. As polyphenols and anthocyanins are well-documented antioxidants exhibiting antibacterial action [64,65,66,67,68], it is reasonable to hypothesize that the capacity to inhibit bacterial growth of the investigated wines may be related to the concentration of these compounds.

The tested products obtained from winemaking could be an alternative to pharmaceuticals and are promising candidates to be used as natural biomaterials, especially in dentistry. In fact, for their antibacterial activity against oral pathogens, the tested products may play not only a role as components of cosmetic products for oral hygiene and ingredients of food supplements and functional foods for oral health, but also in improving physico-chemical properties of biomaterials used in dentistry, thus preventing oral infections and related bone and joint infections [39,40,41]. Moreover, due to their food origin. They are non-toxic, readily available and economical.

## 4. Conclusions

In conclusion, this work represents the first attempt to investigate the changes in the chemical composition of red wine, together with changes in its antibacterial activity, occurring during the winemaking process (including crushing, fermentation and pressing). As far as the chemical composition is concerned, the main changes in the concentration of total polyphenols and total monomeric anthocyanins are positively influenced by the alcoholic fermentation and negatively influenced by the addition of SO_2_. At the end of fermentation, Bonarda red wine reached a TPC and TMA concentration comparable with other red wines at the same stage of winemaking. Metabolite profiling does not change significantly during the winemaking process, and the measured differences can be explained by the various conditions occurring during the process itself. As far as antibacterial activity is concerned, it is possible to synthesize the described results as follows: (i) all the wines collected during winemaking exhibit an antibacterial effect against the tested bacteria strains; (ii) the effect can be considered strain-dependent, as shown by some differences between the bacteria strains; (iii) a great portion of the antibacterial activity may be related to high antioxidant content in the wine; (iv) as a whole, the winemaking process does not seem to exert a significant change on the antimicrobial properties of the analyzed wines, as shown by comparable MIC values.

It is interesting to underline that Croatina Torchiato (CT), which is a wine characterized by a lower economic value and thus destined for a secondary market, proved to be a very rich source of polyphenols. With a chemical composition and antibacterial activity similar to those registered for higher quality wines, and in view of its low cost, CT could be used as a source of new anti-infective agents for the development of innovative biomaterials and coatings for medical devices [69] and could be considered a good alternative to other extracts obtained from tea, cranberry and cocoa [8,9,10,11].

Seen on the whole, our data indicate that polyphenols in red wines are able to exert a significant antibacterial action. Moreover, results of recent experimental studies show that polyphenols express other influential activities/bioactivities, such as anti-inflammatory, antioxidant, and cell membrane stabilizing effects. These findings suggest that polyphenols could be added advantageously and safely to prosthetic biomaterials for counteracting bacterial colonization, without resorting to the addition of toxic chemicals. Thus, in perspective, the incorporation of polyphenols into biomaterials may contribute to a new generation of gentle anti-infective materials, devoid of toxicity and endowed with interesting biological properties.

## Figures and Tables

**Table 1:** List of wine samples, their collection date, step of winemaking process and abbreviation used in this paper.

Wine Sample	Collection Date	Winemaking Step	Abbreviation
Croatina	30 September 2015	Grape crushing, addition of yeast and K_2_S_2_O_5_	CR1
Croatina	2 October 2015	Alcoholic fermentation	CR2
Croatina	6 October 2015	Alcoholic fermentation	CR3
Croatina	8 October 2015	Malolactic fermentation	CR4
Croatina	12 October 2015	Malolactic fermentation	CR5
Croatina	19 October 2015	Malolactic fermentation	CR6
Croatina	26 October 2015	Malolactic fermentation	CR7
Croatina	27 October 2015	Malolactic fermentation	CR8
Barbera	27 October 2015	Wine used to dilute Croatina	BA
Croatina + Barbera before SO_2_ addition	27 October 2015	Addition of Barbera to obtain Bonarda wine	CB1
Croatina + Barbera after SO_2_ addition	27 October 2015	SO_2_ addition	CB2
Bonarda wine (end of fermentation)	30 October 2015	End of fermentation	BO
Croatina Torchiato	8 October 2015	Marc pressing	CT1
Croatina Torchiato	12 October 2015	Fermentation	CT2
Croatina Torchiato	19 October 2015	Fermentation	CT3

**Table 2:** Chromatographic behavior, MS and MSn data of the compounds identified in the 15 wine samples.

Peak Number	Retention Time (RT) (min)	*λ* Max (nm)	*m*/*z*	HPLC-ESI-MSn (% of Base Peak)	Proposed Structure
**Organic and benzoic acids**					
1	12.0	203, 214	191	111 (100)	citric acid
2	28.0	221, 269	169	125 (100)	gallic acid ^(a)^
**Flavonols**					
3	35.4	214, 245, 286	493	331 (100)	laricitrin-hexoside ^(a)^
15	74.9	214, 233, 278	447	401 (100), 285 (100)	kaempferol-hexoside
27	118.3	226, 268, 328	479	316 (100), 317 (30)	myricetin-hexoside
32	121.8	226, 268, 285	463	301 (100)	quercetin-hexoside ^(b)^
35	123.8	226, 312, 348	507	344 (100), 345 (50)	syringetin-hexoside ^(b)^
39	126.1	226, 255, 349	477	301 (100)	quercetin-glucuronide
**Flavan-3-ols**					
4	36.4	207, 269	609	441 (100), 423 (85), 305 (35)	gallocatechin derivative
7	48.0	207, 275	305	179 (100), 221 (80), 219 (80), 261 (70)	gallocatechin ^(c), (d)^
12	69.0	224, 230, 279	289	245 (100), 205 (40), 179 (20)	catechin
20	85.8	228, 279	289	246 (100), 205 (40), 179 (10)	epicatechin ^(c), (d)^
**Tannins**					
5	39.7	219, 278	865	695 (100), 577 (40), 289 (20), 407 (10)	procyanidin trimer
6	44.9	218, 270	331	169 (100)	galloylglucose ^(a), (c), (e)^
9	62.6	279	577	425 (100), 407 (40), 289 (20)	procyanidin dimer
11	67.5	224, 278	577	425 (100), 407 (60), 289 (20)	procyanidin dimer
13	69.6	224, 279	865	695 (100), 577 (80), 407 (25), 298 (20)	procyanidin trimer
16	75.5	278	865	695 (100), 577 (80), 407 (25), 298 (20)	procyanidin trimer
18	79.6	221, 278	577	425 (100), 407 (50), 289 (15)	procyanidin dimer
22	89.8	223, 280	865	695 (100), 577 (40), 793 (40)	procyanidin trimer ^(f)^
**Anthocyanins ^(g)^**					
8	54.5	223, 276	595	443 (100), 425 (80)	cyanidin-6-*O*-coumaroylhexoside
10	64.0	215, 222, 279	465	303 (100)	delphinidin-hexoside
14	74.0	206, 279	449	287 (100)	cyanidin-hexoside
17	79.4	205, 214, 279	479	317 (100)	petunidin-hexoside
19	81.1	226, 280	463	301 (100)	peonidin-hexoside
21	88.0	223, 279	493	331 (100)	malvidin-hexoside
23	93.0	224, 280	507	303 (100)	delphinidin-3-acetylhexoside ^(h), (i)^
24	104.0	206, 219, 280	521	317 (100)	petunidin-3-acetylhexoside
25	107.5	204, 215, 281	517	355 (100)	malvidin-3-glucosylacetaldehyde
26	111.0	224, 280	535	331 (100)	malvinidin-3-acetylhexoside
29	120.0	233, 280	809	357 (100), 519 (80), 547 (20)	malvidin-3-*O*-glucosyl-8-ethyl-epicatechin
30	121.1	232, 280	561	399 (100)	malvidin-3-glucosidepiruvate
31	121.3	230, 283	625	317 (100)	petunidin-3-(6-*O*-coumaroyl)-hexoside ^(l)^
33	122.0	230, 280	609	447 (100), 301 (70)	peonidin-3-O-(C6-coumaroyl)-hexoside ^(l)^
34	122.2	232, 282	639	331 (100)	malvidin-3-coumaroylhexoside
36	124.0	227, 280	707	399 (100)	malvidin-3-*O*-coumaroylglucoside pyruvate
37	124.5	227, 280	707	399 (100)	carboxypyrano-malvidin-3-coumaroylglucoside ^(d)^
38	125.9	226, 355	479	303 (100)	delphinidin-glucuronide
**Stilbenoids**					
28	119.6	-	389	227 (100)	resveratrol-hexoside ^(b)^

^(a)^ Compound not revealed in CR1 sample; ^(b)^ Compound not revealed in BA sample; ^(c)^ Compound not revealed in CR1 and CR2 samples; ^(d)^ Compound not revealed from CT1 to CT3 sample; ^(e)^ Compound not revealed in CT3 sample; ^(f)^ Compound not revealed from CR1 to CR6 sample; ^(g)^ Anthocyanins were revealed in positive ionization mode; ^(h)^ Compound revealed in trace from CR1 to CR6 sample; ^(i)^ Compound not revealed in CT1 and CT2 samples; ^(l)^ Compound revealed in trace in all samples.

**Table 3:** Presence (+) or absence (-) of the identified flavonols, flavan-3-ols, tannins, stilbenoids, benzoic and organic acids in the 15 wine samples.

**Flavonols**	**CR1**	**CR2**	**CR3-CR8**	**BA**	**CB1-CB2**	**BO**	**CT1**	**CT2**	**CT3**
laricitrin-hexoside	-	+	+	+	+	+	+	+	+
kaempferol-hexoside	+	+	+	+	+	+	+	+	+
myricetin-hexoside	+	+	+	+	+	+	+	+	+
quercetin-hexoside	+	+	+	-	+	+	+	+	+
quercetin-glucuronide	+	+	+	+	+	+	+	+	+
syringetin-hexoside	+	+	+	-	+	+	+	+	+
**Flavan-3-ols**	**CR1**	**CR2**	**CR3-CR8**	**BA**	**CB1-CB2**	**BO**	**CT1**	**CT2**	**CT3**
gallocatechin derivative	+	+	+	+	+	+	+	+	+
gallocatechin	-	-	+	+	+	+	-	-	-
catechin	+	+	+	+	+	+	+	+	+
epicatechin	-	-	+	+	+	+	-	-	-
**Tannins**	**CR1**	**CR2**	**CR3-CR8**	**BA**	**CB1-CB2**	**BO**	**CT1**	**CT2**	**CT3**
galloylglucose	-	-	+	-	+	+	+	+	-
procyanidin dimer	+	+	+	+	+	+	+	+	+
procyanidin dimer	+	+	+	+	+	+	+	+	+
procyanidin dimer	+	+	+	+	+	+	+	+	+
procyanidin trimer	+	+	+	+	+	+	+	+	+
procyanidin trimer	+	+	+	+	+	+	+	+	+
procyanidin trimer	+	+	+	+	+	+	+	+	+
procyanidin trimer	-	-	- (CR3-CR6)	+	+	+	+	+	+
**Stilbenoids**	**CR1**	**CR2**	**CR3-CR8**	**BA**	**CB1-CB2**	**BO**	**CT1**	**CT2**	**CT3**
resveratrol-hexoside	+	+	+	-	+	+	+	+	+
**Organic and Benzoic Acids**	**CR1**	**CR2**	**CR3-CR8**	**BA**	**CB1-CB2**	**BO**	**CT1**	**CT2**	**CT3**
citric acid	+	+	+	+	+	+	+	+	+
gallic acid	-	+	+	+	+	+	+	+	+

**Table 4:** Presence (+) or absence (-) of the identified anthocyanins in the 15 wine samples.

Anthocyanin	CR1	CR2	CR3-CR8	BA	CB1-CB2	BO	CT1	CT2	CT3
cyanidin-6-*O*-coumaroylhexoside	+	+	+	+	+	+	+	+	+
cyanidin-hexoside	+	+	+	+	+	+	+	+	+
delphinidin-hexoside	+	+	+	+	+	+	+	+	+
delphinidin-3-acetylhexoside	in trace	in trace	in trace from CR3 to CR6	+	+	+	-	-	+
delphinidin-glucuronide	+	+	+	+	+	+	+	+	+
petunidin-hexoside	+	+	+	+	+	+	+	+	+
petunidin-3-acetylhexoside	+	+	+	+	+	+	+	+	+
petunidin-3-(6-*O*-coumaroyl)-hexoside	in trace								
peonidin-hexoside	+	+	+	+	+	+	+	+	+
peonidin-3-(C6-coumaroyl)-hexoside	in trace								
malvidin-hexoside	+	+	+	+	+	+	+	+	+
malvidin-3-glucosylacetaldehyde	+	+	+	+	+	+	+	+	+
malvidin-3-acetylhexoside	+	+	+	+	+	+	+	+	+
malvidin-3-*O*-glucosyl-8-ethyl-epicatechin	+	+	+	+	+	+	+	+	+
malvidin-3-glucosidepiruvate	+	+	+	+	+	+	+	+	+
malvidin-3-*O*-coumaroylglucoside pyruvate	+	+	+	+	+	+	+	+	+
malvidin-3-coumaroylhexoside	+	+	+	+	+	+	+	+	+
carboxypyrano-malvidin-3-courmaroylglucoside	+	+	+	+	+	+	-	-	-

**Table 5:** Minimal inhibitory concentration (MIC) of each type of dealcoholized wine against two oral *Streptococci* and *S. pyogenes*. Concentration necessary to inhibit 100% of the microbial growth *in vitro*, expressed in v/v % solution.

Delacoholized Wine	*S. mutans* 35176 MIC (v/v %)	*S. salivarius* 11878 MIC (v/v %)	*S. pyogenes* BIO1926 MIC (v/v %)
CR1	60	60	60
CR2	40	40	40
CR3	20	20	40
CR4	20	20	40
CR5	20	20	40
CR6	20	20	40
CR7	20	20	40
CR8	20	20	40
BA	40	40	40
CB1	20	20	40
CB2	20	20	40
BO	20	20	20
CT1	20	20	20
CT2	20	20	20
CT3	20	20	20

## Abbreviations

The following abbreviations are used in this manuscript:

CLSI	Clinical and Laboratory Standards Institute
CGE	Cyanidin-3-glucoside equivalents
GAE	Gallic acid equivalents
MIC	Minimum inhibitory concentration
NCCLS	National Committee for Clinical Laboratory Standards
TMA	Total monomeric anthocyanins
TPC	Total polyphenol content
YAN	Yeast assimilable nitrogen
